# Longitudinal analysis of refraction and ocular biometrics in preschool children with early-onset high myopia

**DOI:** 10.1038/s41598-023-50004-8

**Published:** 2023-12-18

**Authors:** Hae Ri Yum, Shin Hae Park, Sun Young Shin

**Affiliations:** 1 Department of Ophthalmology, College of Medicine, Eunpyeong St. Mary’s Hospital, The Catholic University of Korea, Seoul, Republic of Korea; 2 Department of Ophthalmology, College of Medicine, Seoul St. Mary’s Hospital, The Catholic University of Korea, Seoul, Republic of Korea

**Keywords:** Diseases, Risk factors

## Abstract

We investigated changes in refraction and ocular biometrics in preschool children with early-onset high myopia. Sixty eyes of 60 children with a mean follow-up time of 58.5 months were included in this study. At baseline, mean age of children was 55.6 ± 13.1 months, mean spherical equivalent (SE) was − 8.59 ± 2.66 D, and 25.64 ± 1.16 mm for axial length (AL). The total annual rate of myopic progression and axial elongation were − 0.37 ± 0.39 D/year and 0.33 ± 0.18 mm/year, respectively. During follow-up period, there was a trend toward less myopic progression and axial elongation over time. Of the total participants, 24 children (40%) were in the myopia progression group and the remaining 36 children (60%) were in the myopia stability group. In multiple linear regression analysis, baseline SE and AL were independently associated with myopic progression, while age, sex, and baseline AL-to-CR ratio were not related to myopic progression. According to the model, more myopic SE (β = − 0.186, *P* = 0.035) and longer AL (β = − 0.391, *P* = 0.008) at baseline were significantly associated with myopic progression. Myopia progression in preschoolers with high myopia tended to be relatively modest, with 60% of subjects exhibited myopic stability. Higher myopic SE, and longer AL at baseline were associated with myopic progression in preschool children with high myopia.

## Introduction

High myopia is defined as a spherical equivalent (SE) refractive error over − 6.0 diopters (D), and/or an ocular axial length (AL) ≥ 26 mm^1,2^. The prevalence of high myopia has increased worldwide in the past years. It has been recently reported to affect 3–10% of the worldwide population, and more than 10% in some Asian countries^3–7^. High myopia must be closely monitored because it is a significant public health concern due to its association with a higher risk of high-myopia related irreversible ocular complications related to vision loss and reduced quality of life^8^. Each 1 D of myopic progression increases the prevalence of myopic maculopathy, retinal detachment, and open angle glaucoma by 58%, 30%, and 20%, respectively^9^. Overall, the cumulative risk of visual impairment increases from 24 to 31% per 1 D of myopic progression^9^. Earlier myopia onset and higher myopic refraction at baseline are two main risk factors for high myopia^10^. Especially, children with early-onset high myopia exhibit longer period of myopic progression and potentially higher degree of myopia until refractive stabilization^11,12^. Therefore, it is important to monitor myopic progression in children with early-onset high myopia. However, most studies of myopia progression have focused on school-aged children, and there is a lack of research on long-term changes in preschoolers with high myopia. In this study, we aimed to analyze longitudinal changes of refraction and ocular biometrics in preschool children with early-onset high myopia.

## Methods

### Subjects

Children aged ≤ 6 years with a spherical equivalent (SE) refractive error of -6.0 D or greater in one or both eyes were included in this study. These children had their initial visit to the pediatric outpatient eye clinic at a tertiary institution between March 2015 and November 2019 and were followed for more than 3 years. If both eyes had high myopia, data from the right eye was chosen. Children with astigmatism (more than − 2.0 D) and/or other ocular diseases such as congenital cataract, glaucoma, or corneal or retinal diseases, and/or a history of ocular trauma or ocular surgery were excluded from the study. The medical records were retrospectively reviewed, and patient information was anonymized and de-identified prior to analysis. Parents of the children were asked whether they wore glasses or contact lenses to correct high myopia more than − 6.0 D. Children were classified into myopic progression group (progression of myopic refraction of ≥ 0.5 D/year) and myopic stability group (change of refraction of < 0.5 D/year). This study was approved by the Catholic University of Korea Institutional Review Board (KC16RISI0443) and adhered to the tenets of the Declaration of Helsinki. The institutional review board waived the requirement for obtaining informed consent from the participants.

### Outcome measures and statistical analysis

Refraction was measured using a Huvitz HRK-7000A® auto refractor keratometer (Coburn Technologies, South Windsor, CT, USA) after cycloplegia and calculated as SE. The cycloplegic regimen consisted of three drops of cyclopentolate hydrochloride (10 mg/mL, OcuCyclo®, Samil, Republic of Korea) administered approximately 10 min apart. Cycloplegic autorefraction measurements were performed at least 30 min after the third drop of cyclopentolate had been administered. Axial length (AL) was measured using ocular biometry (IOL Master 700; Carl Zeiss Meditec, Inc., Dublin, CA, USA). Five measurements were obtained for each eye. AL was calculated using the automated system included with the equipment. All statistical analyses were performed using the Statistical Package for the Social Sciences (SPSS) software version 18.0 (SPSS, Inc., Chicago, IL, USA). The Kolmogorov–Smirnov test was used to evaluate data distributions. The Pearson’s chi-squared test was used to analyze group differences for categorical data, and paired t-test and two-sample t-tests, were used to analyze group differences for continuous data. Multiple linear regression analysis was performed to assess the association of myopic progression with age, sex, and baseline SE, AL, and AL-to-CR ratio. Odds ratios (ORs) with 95% confidence intervals (CIs) were calculated. *P*-values of less than 0.05 were considered statistically significant.

## Results

A total of 60 eyes of 60 children were analyzed in this study. The mean age was 55.6 ± 13.1 months, mean SE was − 8.59 ± 2.66 D, and mean AL was 25.64 ± 1.16 mm at the initial visit (Table 1). There was a trend toward less myopic progression and axial elongation during 3 years of follow-up. At the first year, the mean SE was − 9.12 ± 2.73 D, and the mean AL was 26.11 ± 1.22 mm. The annual rates of myopic progression and axial elongation were − 0.46 ± 0.72 D/year and 0.41 ± 0.24 mm/year, respectively. At the second year, the mean SE was − 9.37 ± 2.60 D, and the mean AL was 26.39 ± 1.27 mm. The annual rates of myopic progression and axial elongation were − 0.28 ± 0.56 D/year and 0.31 ± 0.21 mm/year, respectively. At the third year, the mean SE was − 9.62 ± 2.67 D, and the mean AL was 26.66 ± 1.35 mm. The annual rates of myopic progression and axial elongation were − 0.26 ± 0.59 D/year and 0.28 ± 0.16 mm/year, respectively (Table 1). The total annual rate of myopic progression and axial elongation were − 0.37 ± 0.39 D/year and 0.33 ± 0.18 mm/year, respectively.

**Table 1:** Demographic characteristics of the children with early-onset high myopia and changes of refractive error and ocular biometric examination.

	Initial visit	1 yr	2 yr	3 yr
Age (mo)	55.6 ± 13.1	69.5 ± 12.7	80.8 ± 12.7	92.1 ± 11.9
SE (D)	− 8.59 ± 2.66	− 9.12 ± 2.73	− 9.37 ± 2.60	− 9.62 ± 2.67
AL (mm)	25.64 ± 1.16	26.11 ± 1.22	26.39 ± 1.27	26.66 ± 1.35
CCT (mm)	529.4 ± 28.9	531.7 ± 29.5	535.0 ± 32.2	538.2 ± 31.0
ACD (mm)	3.59 ± 0.22	3.66 ± 0.24	3.72 ± 0.20	3.73 ± 0.20
LT (mm)	3.51 ± 0.15	3.45 ± 0.17	3.40 ± 0.15	3.39 ± 0.16
Mean K (D)	43.75 ± 1.25	43.64 ± 1.05	43.76 ± 1.05	43.68 ± 1.12
CR (mm)	7.72 ± 0.22	7.74 ± 0.19	7.72 ± 0.18	7.73 ± 0.20
AL-to-CR ratio	3.33 ± 0.12	3.36 ± 0.08	3.41 ± 0.10	3.45 ± 0.12
Change of SE (D/yr)		− 0.46 ± 0.72	− 0.28 ± 0.56	− 0.26 ± 0.59
*P*-value		0.057	0.164	
Change of AL (mm/yr)		0.41 ± 0.24	0.31 ± 0.21	0.28 ± 0.16
*P*-value		0.002	0.236	

SE, spherical equivalent; D, diopter; AL, axial length; CCT, central corneal thickness; ACD, anterior chamber depth; LT, lens thickness; K, keratometer; CR, corneal radius of curvature.

The other ocular biometric measurements were 3.59 ± 0.22 mm for anterior chamber depth (ACD), 3.51 ± 0.15 mm for lens thickness (LT), 7.72 ± 0.22 mm for corneal radius of curvature (CR), and 3.33 ± 0.12 for AL-to-CR ratio at the baseline (Table 1). The ACD and AL-to-CR ratio exhibited a pattern of incremental change, approximately 0.14 mm and 0.12, respectively (both *P* < 0.001). Meanwhile, the LT displayed a trend of decreasing change, approximately 0.12 mm, over three-year periods (*P* < 0.001). Unlike ACD, AL-to-CR ratio, and LT, CR remained stable throughout the study (*P* = 0.178).

Twenty-four of the children (40%) exhibited myopic progression 0.5 D/year or more, while thirty-six of the children (60%) displayed myopic progression less than 0.5 D/year during the follow-up period (Table 2). Significant myopia progression was observed in the myopic progression group compared to the myopic stability group (−0.73 ± 0.23 D/year, vs. − 0.13 ± 0.27 D/year, *P* < 0.001). Also, the rate of axial elongation was significantly steeper in the myopic progression group compared to the myopic stability group (0.42 ± 0.17 mm/year, vs. 0.27 ± 0.16 mm/year, *P* = 0.002). However, there were no significant differences in the baseline characteristics including age, sex, parental high myopia, refraction, and biometric parameters between two groups.

**Table 2:** Comparisons of clinical characteristics at baseline between myopic progression and stability group.

	Myopic progression (N = 24)	Myopic stability (N = 36)	*P-*value
Age (month)	55.8 ± 13.1	55.5 ± 13.3	0.930
Sex (F:M)	12:12	16:20	0.673
Parental high myopia	14	13	0.090
Initial SE (D)	− 8.38 ± 3.15	− 8.74 ± 2.31	0.612
Initial AL (mm)	25.83 ± 0.97	25.52 ± 1.28	0.326
Initial CCT (mm)	531.4 ± 26.7	521.5 ± 27.4	0.388
Initial ACD (mm)	3.65 ± 0.22	3.56 ± 0.22	0.256
Initial LT (mm)	3.44 ± 0.18	3.54 ± 0.12	0.070
Initial mean K (D)	43.49 ± 1.07	43.91 ± 1.34	0.289
Initial CR (mm)	7.76 ± 0.19	7.69 ± 0.24	0.311
Initial AL-to-CR ratio	3.32 ± 0.13	3.33 ± 0.12	0.919
Follow up period (month)	59.8 ± 31.9	57.6 ± 32.6	0.802
Total change of SE (D/yr)	− 0.73 ± 0.23	− 0.13 ± 0.27	< 0.001
Total change of AL (mm/yr)	0.42 ± 0.17	0.27 ± 0.16	0.002

SE: spherical equivalent, D: diopter, AL: Axial length: CCT: central corneal thickness, ACD: anterior chamber depth, LT: lens thickness, K: keratometer, CR: corneal radius of curvature.

In multiple linear regression analysis, baseline SE and AL were independently associated with myopic progression, with a R2 of 0.365, while age, sex, and baseline AL-to-CR ratio were not related to myopic progression (Table 3). According to the model, more myopic SE (β = − 0.186, *P* = 0.035) and longer AL (β = − 0.391, *P* = 0.008) at baseline were significantly associated with myopic progression.

**Table 3:** Multivariable linear regression analysis of variables with myopic progression.

Variables	Coefficient (95% CI)	Standardized coefficient	*P*-value
Age (months)	0.005 (− 0.004, 0.014)	0.104	0.617
Sex	0.109 (− 0.083, 0.301)	0.094	0.574
Baseline SE (D)	− 0.186 (− 0.270, − 0.102)	− 0.617	0.035
Baseline AL (mm)	− 0.391 (− 0.528, − 0.254)	− 0.626	0.008
Baseline AL-to-CR ratio	− 0.776 (− 2.741, 1.189)	− 0.114	0.696

R2 = 0.365.

SE: spherical equivalent, AL: axial length, AL/CR ratio: axial length/corneal radius ratio, 95% CI: 95% confidence interval.

## Discussion

This study investigated longitudinal changes of refractive error and ocular biometrics in preschool children with early-onset high myopia over a 3-year period. The total annual rate of myopic progression and axial elongation were − 0.37 ± 0.39 D/year and 0.33 ± 0.18 mm/year, respectively (Table 1). Yin et al. reported that myopia progressed by − 0.59 ± 0.47 D/year in children with preschool myopia^13^. The initial median age and SE was 5.12 years and − 3.00 D in the study. Guo et al. reported that axial length increased 0.24 mm/year in boys and 0.32 mm/year in girls from 3 to 6 years of age in the Shenzhen Kindergarten Eye study^14^. The mean age was 5.0 years, and the average SE was 1.38 D in the study. Our study revealed that the progression of myopia in preschoolers with early-onset high myopia was slower than that in those with an average of – 3 D of myopia^13^. Human and animal studies suggest that the development and progression of myopia are controlled by environmental, genetic, and possibly physiological factors. We hypothesized that the mechanism of early-onset high myopia might be different from that of other myopia in school-aged children. Genetic factors might be the main cause leading to early-onset high myopia, and then the emmetropization process activate to stabilize eye growth. It is also estimated to be relatively less affected by environmental factors.

Overall, a 1 mm increase in axial length would lead to approximately 1.40 D of myopia in the current study. Guo et al. reported that an increase of a 1 mm in axial length was associated with only 0.45 D of myopic progression in 3- to 6-year-old preschoolers^14^. The NICER Study reported that the ∆SE/∆AL for 6- to 7-year-old myopic children was 1.22 D/mm and the ∆SE/∆AL for 6- to 7-year-old emmetropic children was 0.45 D/mm, respectively^15^. The reason why ∆SE/∆AL was higher in our study (1.40 D/mm) than that found in another study (0.45 D/mm) of the similar age group, was that the current study targeted children with early-onset high myopia, while the mean SE in other studies was 1.37 D^14^. Another study in Shanghai of 1856 children aged 7–9 years found that ∆SE/∆AL was 0.83, 1.74, and 1.83 D/mm in children with emmetropia, incipient myopia, and persistent myopia, respectively^16^. Jones et al.^17^ reported that 0.5 mm of axial elongation would result in approximately 1.50 D of myopia in children between the ages of 6 and 14 years. The present study focused on preschoool children under the age of 6, so ∆SE/∆AL was relatively lower compared to the results of the study conducted on school children. This finding is consistent with a previous study on myopia, where a 1 mm increase in AL corresponded to more myopic progression in SE for older children^18^.

Our study showed gradually decreased myopic progression and axial elongation over 3-year study period, which was like previous studies^16,18^. The previous study reported that AL elongation decreased to the order of 0.14 mm/year from 3 to 6 years of age^14^. In the current study, myopic progression decreased from − 0.46 D/year to − 0.26 D/year, and AL growth decreased from 0.41 to 0.28 mm/year during 3-year period. It has been documented that the progression of myopia and axial elongation exhibits its highest rates in the year immediately preceding the onset of myopia, subsequently decelerating following the onset^19,20^. During normal eye growth, the development of the crystalline lens is characterised by thinning, flattening and decreasing power to maintain emmetropia by compensating for eye growth. In our study, the lens became progressively thinner over time. We speculated that this compensatory effect reduced the rate of myopia progression and axial elongation over time^21^. It is speculated that high myopia with axial elongation creates a peripheral myopic defocus in the retina, which inhibits myopia progression. The visually guided feedback mechanism adjusts the rate of axial growth of the eye to the optical focal length^22^. Several studies discovered that myopic defocus regulates the growth of the eye^23,24^.

In our study, 40% of children showed myopic progression, while 60% exhibited myopic stability over a 3-year study period. The previous study reported that 45% of children showed myopic progression, and 55% of children showed stability or regression in children with infantile-onset myopia, which is consistent with our results^25^. There were no significant differences in baseline characteristics, including age, sex, parental high myopia, refraction, and biometric parameters, between children who exhibited myopic progression of 0.5 D/year or more and children who displayed myopic progression of less than 0.5 D/year during the follow-up period. In the multiple linear regression analysis, myopic progression was significantly associated with more severe myopic refraction and longer AL at baseline. The previous study analyzed factors affecting AL changes in children with high myopia: younger age and longer baseline AL had significant association with AL changes^25^.

In addition, the trends of ACD, LT, and CR change are like previous studies^14,17,26^. ACD increased approximately 0.14 mm and LT decreased 0.12 mm from 3 to 6 years of age in this study. Unlike ACD and LT, CR remained stable throughout the study. Guo et al. reported ACD increased with age from 3 to 6 years of age at a rate of 0.10 mm/year^14^. The COMET study also demonstrates comparable results in terms of increases in anterior chamber depth^26^. In our study, the mean LT was 3.51 mm at initial visit, which was thinner than previous study^17,27^. In this study, it is not known whether the thinning of lens thickness affected axial length growth or whether lens thinning occurred as a compensatory mechanism of axial elongation. As lens becomes thinner, the lens power declines its ability to compensate for AL elongation. Lisa et al. reported a decrease in lens thickness of approximately 0.14 mm over an age range of 6 to 11 years^17^. Donald et al. reported that average lens thinned in the early childhood from 3.59 mm at age 6 years to 3.43 mm at age 10 years^27^. The AL-to-CR ratio was higher in this study compared to that of general children with same age (3.33 ± 0.12 vs. 2.88 ± 0.06)^14^. The cutoff value for AL-to-CR ratio as a myopia detection is reported approximately > 3.00^28,29^. Furthermore, AL-to-CR ratio increases gradually with age and increases at the rate of approximately 0.04 to 0.05 for each 1D of myopia^28^. The subjects in this study had an average initial myopia of approximately − 8.6 D, which is approximately − 10 D more than the average of 1.4 D for the same age, resulting in an increase in AL to CR ratio of approximately 0.45 compared to previous studies.

This study has several limitations. It is a retrospective study from a single institution. The prevalence of high myopia in preschool children is low, resulting in a relatively small number of cases in this study. In addition, within the limitations of this study, no clinically apparent differences were observed between the groups characterized by myopia progression and stability. It is conceivable that other clinical and genetic factors may have influenced the progression of myopia in children with high myopia. Nevertheless, the study has notable strengths, particularly the inclusion of a cohort comprised of highly myopic preschool children, a demographic group underrepresented in the literature. In addition, the study benefits from a substantial mean follow-up duration of 58.5 months, which allows for a comprehensive examination of longitudinal changes in both refraction and ocular biometrics.

In conclusion, myopia progression in preschoolers with high myopia tended to be relatively modest, with 60% of subjects exhibited myopic stability. The findings of our study indicate that a higher myopic SE, and a longer AL are associated with myopic progression in preschool children with high myopia. Further large-scale longitudinal studies are necessary to determine changes in ocular structures and related factors of myopic progression in children with early-onset high myopia.

## Data Availability

The datasets utilized and/or examined in the course of this study are available from the corresponding author upon a reasonable request, in accordance with established medical research protocols.
